# Diphtheria outbreak in Jakarta and Tangerang, Indonesia: Epidemiological and clinical predictor factors for death

**DOI:** 10.1371/journal.pone.0246301

**Published:** 2021-02-04

**Authors:** Eggi Arguni, Mulya Rahma Karyanti, Hindra Irawan Satari, Sri Rezeki Hadinegoro

**Affiliations:** 1 Department of Child Health, Faculty of Medicine, Public Health and Nursing, Universitas Gadjah Mada, Yogyakarta, Indonesia; 2 Department of Pediatric, Faculty of Medicine, University of Indonesia, Jakarta, Indonesia; Meyer Children’s University Hospital - University of Florence, ITALY

## Abstract

**Background:**

In 2017, a diphtheria outbreak occurred in several provinces in Indonesia. The aim of this study was to identify predictors of mortality outcome of pediatric patients with clinical diphtheria.

**Methods:**

A retrospective cohort study was conducted using patient medical records at five referral hospitals in the Province of Jakarta and one in Tangerang District, Banten Province during January 2017 to 31 August 2018. All children in the age group of 1–18 years old discharged with diagnosis of clinical diphtheria formed the study group. All anonymized patient data were evaluated for demographic issues, clinical features, immunization status, complication, laboratory profiles and outcome.

**Results:**

A total of 283 patients with clinical diphtheria were included in the study group with case fatality rate of 3.5%. All mortal patients had the complication of myocarditis. Regression analyses revealed factors for predicting mortality. Incomplete primary diphtheria toxoid immunization, stridor, bull neck, leukocytosis ≥15 x10^9^ cells/L and thrombocytopenia ≤150 x10^9^ cells/L in each combination for 2 predictors modeling were correlated with death.

**Conclusions:**

We report key predictors of mortality in pediatric patients with clinical diphtheria. The presence of these features when admitted to the hospital must be taken into account, because they can lead to fatal outcome.

## Introduction

Diphtheria is an infectious disease caused by *Corynebacterium diphtheriae* (*C*. *diphtheriae*) with high mortality rates. The world faces the fact that globally there has been a significant decrease in diphtheria cases since a vaccine was included in most countries’ national immunization programs in the 1970s. Nevertheless, this disease has not completely disappeared from the earth. Several countries still have reported diphtheria cases between 1970–1999, namely the Russian Republics, Nigeria, India, Bangladesh, Vietnam and several places in South America, including Brazil [1, 2]. In Indonesia, even though the diphtheria vaccine has been introduced in the national expanded program on immunization (EPI) since 1977, cases were reported sporadically from several provinces. The Ministry of Health of the Republic of Indonesia reported increasing cases since 2006 [3]. In 2016, 4 diphtheria cases with 1 fatal case were reported in Jakarta Province. In the period of January–December 2017, a total of 939 cases with 44 fatal cases were reported from 170 districts, in 30 provinces in Indonesia, then it was announced as a national outbreak [4].

One of the possible reasons why this outbreak occurred was the low coverage of diphtheria vaccination and its unequal distribution throughout Indonesia. National Diphtheria Pertussis Tetanus vaccination (DPT3) coverage reached 85% in 2013, but it was not equal in every village and district. Demographic and socio-economic aspects, age less than 5 years old or more than 40 years old, low socio-economic status, unvaccinated or incompletely vaccinated, late admission to hospital, delayed antitoxin treatment, myocarditis and airway obstruction are the risks, and at the same time, predictor factors for death [5–7]. The aim of this study was to identify the predictor factors for death from clinical diphtheria in pediatric cases during the 2017–2018 diphtheria outbreak in Jakarta Province and Tangerang District, Banten Province.

## Methods

### Study setting and population

This was a multicenter retrospective cohort study using anonymized medical records at referral hospitals for diphtheria, namely Dr. Cipto Mangunkusumo General Hospital, Dr. Sulianti Saroso Infectious Diseases Hospital, Persahabatan Hospital, Fatmawati Hospital and Koja Hospital in Jakarta Province; and Tangerang District Hospital in Banten Province. The study had received ethical approval from the Dr. Cipto Mangunkusumo General Hospital and the Medical Faculty University of Indonesia review board (0297/UN2.F1/ETIK/2018). All pediatric patients aged 1–18 years old who were admitted to the hospitals with diagnosis of suspected diphtheria or probable diphtheria in the period of January 1 to August 31, 2018 were included. Suspected diphtheria was defined as a person with an illness characterized by laryngitis, pharyngitis or tonsillitis, and adherent membrane of the tonsils, pharynx and/or nose. Probable diphtheria was defined as suspected diphtheria with at least one of sign and symptom: contact with confirmed case within less than two weeks, incomplete diphtheria vaccination including booster, live in endemic area, stridor, bull neck, submucosal bleeding or petechiae, heart failure, renal failure, myocarditis or death. Clinical diphtheria was defined as the final diagnosis when suspected or probable diphtheria patient was discharged from hospital without culture proven of *C*. *diphtheriae*. We excluded patients leaving the hospital against medical advice, patients with congenital heart disease or who had been diagnosed as myocarditis previously. Cultures were done at the Indonesia National Institute of Health Research and Development.

### Data collection and statistical analysis

Sociodemographic and clinical data including age, gender, duration of illness at presentation, clinical features, vaccination history, were extracted from medical records. Descriptive statistics were presented as frequency (percentage) for categorical variables. Mean ± standard deviation (SD) and median (minimum and maximum) were reported for continuous variables. Comparison between groups were performed using Chi-squared test for categorical data. Survivors and non-survivors were compared using univariate analysis to identify predictors having a significant association with mortality. Odds ratio (OR) with 95% confidence interval (CI) was computed for the significant variables. All variables found to be significant in bivariate analysis (*p*<0.25) were subjected to multiple logistic regression analysis to determine possible associations of predictor variables with outcome of death and to obtain OR before and after adjustment with significant predictors (*p*<0.05). The statistical analysis was done using SPSS software v. 20.0 (SPSS Inc., Chicago, IL, USA).

## Results

During the period of January 2017 –August 2018, 400 patients met the criteria for inclusion, which consisted of 389 patients admitted with diagnosis of suspected diphtheria and 11 with diagnosis of probable diphtheria. The number of patients with diagnosis of clinical diphtheria who were discharged from the hospitals were 304 (75.8%) and 96 (24.4%) were discharged with other diagnoses (Table 1).

**Table 1:** Number of patients based on hospital.

Site study	Admission Diagnosis		Total	Discharge Diagnosis	
	Suspected diphtheria	Probable diphtheria		Clinical diphtheria (%)*	Acute tonsillitis (non-diphtheria), or others (%)
Dr. Cipto Mangunkusumo Hospital	7	2	9	9 (100)	0
Dr. Sulianti Saroso Infectious Diseases Hospital	281	1	282	215 (76.2)	67 (23.8)
Fatmawati Hospital	20	2	22	7 (31.8)	15 (68.2)
Persahabatan Hospital	30	0	30	16 (53.3)	14 (46.7)
Koja Hospital	7	0	7	7 (100)	0
Tangerang District Hospital	44	6	50	50 (100)	0
Total	389	11	400	304 (76)	96 (24)

* Percentage of the total patients admitted with suspected and probable diphtheria diagnosis.

**Fig 1:**
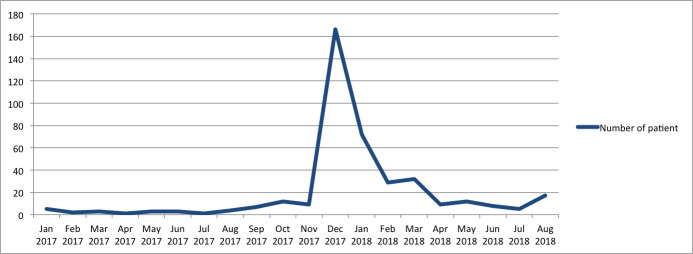
Diphtheria cases admitted to hospital by month.

The number of incidences shows that there was an increase in the number of suspected and probable diphtheria cases admitted to the hospitals in Jakarta Province and Tangerang District, Banten Province in August 2017, with the peaks occurring in December 2017 and January 2018 (Fig 1, S1 Data).

Final discharge diagnosis of clinical diphtheria was confirmed in 304 patients Five patients were discharged against medical advice and 16 patients were reported to be admitted in two hospitals (double-recording). This double-recording of patients was recorded at one of the main hospitals. From the entire process, the final 283 patients were further analyzed (S2 Data).

Table 2 indicates the clinical and laboratory characteristics of the clinical diphtheria cases. During January 2017 and August 2018, we recorded 10 deaths (case fatality rate, CFR 3.5%). There was a larger proportion of male (58.3%) than female patients (41.7%). The highest incidence of diphtheria was found among 1–9 years age group, which consisted of 1–4 years age group (28.6%) and 5–9 years age group (37.8%).

**Table 2:** Characteristics of research subjects (n = 283).

Characteristic	Patients (n)	Percentage (%)
Male sex	165	58.3
Age group (years)		
1–4	81	28.6
5–9	107	37.8
10–14	52	18.4
15–18	43	15.2
Death	10	3.5
Incomplete primary diphtheria toxoid immunization	71	25.1
Fever	270	95.4
Sore throat	260	91.9
Hoarse voice	9	3.2
Stridor	54	19.1
Bull neck	68	24.0
Cough	152	53.7
Nasal discharge	91	32.2
Evidence of myocarditis	45	15.9
Pseudomembrane	265	93.6
Lymphadenopathy	107	37.8
Received diphtheria antitoxin	213	75.3
Onset day of received antitoxin, median (minimum-maximum)	4 (0–16)	NA
Length of stay, median day (minimum-maximum)	9 (1–26)	NA
Onset day of hospital admission, median (minimum-maximum)	3 (0–9)	NA
Admission temperature, median ^0^C (minimum-maximum)	37.0 (36.0–39.9)	NA
Pre-admission treatment with antibiotic	77	27.2
Hb, g%, mean (±SD)	12.3 (±1.3)	
WBC count, cells/L, median (minimum-maximum)	13.1x10^9^ (1.7x10^9^-37.3 x10^9^)	NA
Platelet count, cells/L, median (minimum-maximum)	282.500x10^9^ (11 x10^9^-765 x10^9^)	NA
*C*. *diphtheriae* isolated	46	16.3
No data of isolated pathogen	227	80.2

**Note**: Data include the number of patients (N) unless indicated. NA, not applicable; Hb, hemoglobin; WBC, white blood cell.

The trend in diphtheria declined among children in the higher age group. Data on the history of contact and travel were not sufficiently available in the medical records. Twenty-five percent (25.1%) of the children had incomplete primary diphtheria toxoid vaccination (lack of any or all DPT-1, DPT-2, DPT-3 before 1 year old). Considering together the booster diphtheria toxoid immunization after 1 year old to 18 years old, 251 children (88.7%) have incomplete immunization status.

**Fig 2:**
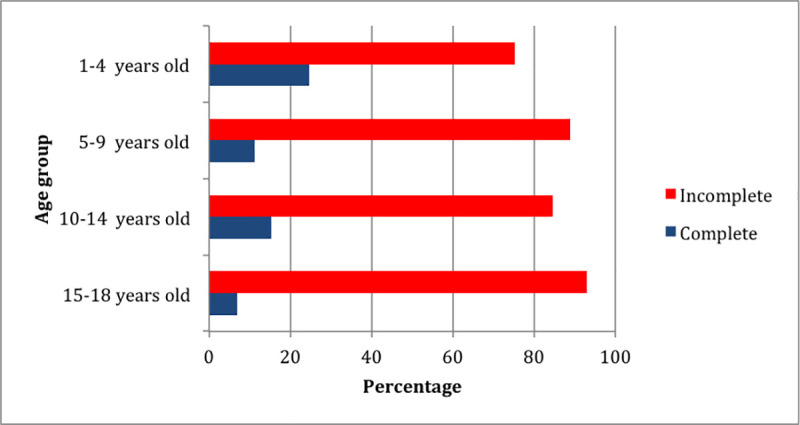
The association between age groups and immunization status.

Fig 2 shows the association between age groups and immunization status. Most patients in all age groups had incomplete immunization history. Almost all patients in the 15–18 year age group did not have complete immunization history (93%) (S3 Data).

Data from results of throat swab culture examined by the National Institute of Health Research and Development of the Republic of Indonesia were obtained through the Jakarta Province Health Office and from the surveillance units in each hospital. From 283 throat swab samples, 46 cultures were found positive *C*. *diphtheriae*, 202 cultures showed negative results and 35 sample results were not available. The most common biotype isolated was intermedius (36.9%), followed by mitis (28.3%) and gravis biotypes (19.6%). Belfanti biotype was not identified in this research. Seven samples (15.2%) were unidentified (S2 Data).

In order to examine factors associated with death, bivariate and multivariate logistic regressions analyses were used. Table 3 presents the association between epidemiological, clinical and laboratory variables with death outcome.

**Table 3:** Predictive factors suspected to affect mortality outcome (n = 283).

Characteristic	Death (n = 10)		Survivor (n = 273)		OR (95%CI)	*p* value*
	n	%	n	%		
Male sex	8	80	157	57.5	2.955 (0.616–14.177)	0.202
Age, median year (min-max)	6 (4–13)		7 (1–18)		NA	0.386
Incomplete primary immunization	7	70	64	23.4	7.620 (1.915–30.324)	0.003
Fever	10	100	260	95.2	NA	1.000
Sore throat	10	100	250	91.6	NA	1.000
Hoarse voice	2	20	7	2.6	9.500 (1.698–53.141)	0.035
Stridor	8	80	46	16.8	19.739 (4.059–95.983)	<0.001
Bull neck	9	90	59	21.6	32.644 (4.054–262.868)	<0.001
Cough	5	50	147	53.8	0.857 (0.243–3.029)	1.000
Nasal discharge	4	40	87	31.9	1.425 (0.392–5.180)	0.732
Myocarditis	10	100	35	12.8	NA	<0.001
Pseudomembrane	10	100	255	93.4	NA	1.000
Lymphadenopathy	7	70	100	36.6	4.037 (1.021–15.961)	0.045
Absent of antitoxin treatment	0	0	70	25.6	NA	0.127
Onset day of antitoxin treatment ≥2 day on admission**	0	0	30	14.8	NA	0.364
Onset day of hospital admission, median	4 (2–7)		3 (0–9)		NA	0.025
WBC count ≥15x10^9^ cells/L^#^	8	80	88	33.1	8.091 (1.683–38.906)	0.004
Platelet count ≤150 x10^9^ cells/L^#^	7	70	10	3.8	59.733 (13.425–265.783)	<0.001
*C*. *diphtheriae* isolated***	6	100	40	80.0	NA	0.578

**Note**: Data include the number of research subjects (n) unless otherwise stated concerning other measurements than those included in the column on characteristics. OR: odds ratio; NA: not applicable; WBC: white blood cell.

* Fisher’s exact test.

** Patients who obtained antitoxin treatment (n = 213; died = 10; survived = 203).

***From swab culture available patient (n = 56), culture positive = 46.

^#^ 266 survivors who had complete WBC and platelet counts.

After bivariate logistic regression analysis, we found that incomplete primary diphtheria toxoid vaccination, and clinical symptoms including hoarse voice, stridor, bull neck, lymphadenopathy, myocarditis complications, onset day of hospital admission, leukocytosis ≥15 x10^9^ cells/L and thrombocytopenia ≤150 x10^9^ cells/L affected mortality (*p*<0.05). Predictors suspected to affect mortality that achieved statistical significance of *p*<0.25 in the bivariate analyses were retained in the multiple logistic regression analysis, as unadjusted OR determinants of death (Table 4, Model 1). Myocarditis complication and absence of antitoxin treatment were excluded since all of the death cases have myocarditis complication, and all of the death cases received antitoxin.

**Table 4:** Unadjusted (Model 1) and adjusted (Models 2–8) odds ratios as determinants for death in clinical diphtheria.

	Unadjusted OR (Model 1)	Adjusted OR (Model 2)	Adjusted OR (Model 3)	Adjusted OR (Model 4)	Adjusted OR (Model 5)	Adjusted OR (Model 6)	Adjusted OR (Model 7)	Adjusted OR (Model 8)
Male sex	2.955 (0.616–14.18)	3.733 (0.472–29.52)	3.807 (0.284–50.94)	---	---	---	---	---
Incomplete primary immunization	7.620** (1.915–30.32)	8.079* (1.438–45.40)	12.49* (1.615–96.64)	---	---	8.182* (1.553–43.099)	7.885** (1.832–33.932)	6.603** (1.553–28.069)
Hoarse voice	9.500* (1.698–53.14)	3.2 (0.214–47.89)	1.614 (0.0660–39.51)	---	---	---	---	---
Stridor	19.74*** (4.059–95.98)	11.83** (2.156–64.97)	10.50* (1.563–70.60)	---	---	---	20.247*** (4.005–102.367)	---
Bull neck	32.64** (4.054–262.9)	---	24.38** (2.161–275.1)	17.650* (1.984–156.999)	7.595* (1.497–38.537)	---	---	29.739** (3.628–243.790)
Lymphadenopathy	4.037* (1.021–15.96)	2.687 (0.468–15.45)	---	---	---	---	---	---
Leukocytosis ≥15 x10^9^ cells/L	8.091** (1.683–38.9)	5.126 (0.916–28.68)	4.852 (0.806–29.21)	---	31.627** (3.855–259.449)	63.464*** (12.328–326.722)	---	---
Thrombocytopenia ≤150 x10^9^ cells/L	59.73*** (13.42–265.8)	---	---	31.313*** (6.264–156.532)	---	---	---	---
Nagelkerke R^2^		0.565	0.456	0.508	0.365	0.475	0.343	0.356

**Note**: Exponentiated coefficients = odds ratio (OR); 95% confidence interval (CI) in parentheses

* *p*<0.05

** *p*<0.01

*** *p*<0.00.

We tested all of the candidate predictors to see whether they have the potential of interfering with each other in causing death, and excluded those to be analyzed in the same adjusted model (Table 4, Models 2–9, S4 Data). Males were about 4 times and patients with hoarse voice were 2–3 times more likely to die, but the results were not statistically significant (Table 4, Models 2 and 3). In the unadjusted model, clinical signs of stridor and bullneck have strong association with death, but might be interfered when adjusted by the variable of thrombocytopenia (Models 2 and 3). Notably the variables of bull neck and lymphadenopathy might have interfered with each other in causing death, thus we retained the variable of bull neck for the adjusted Model 3, since bull neck pathophysiologically makes more sense. Both leukocytosis ≥15x10^9^ cells/L and thrombocytopenia ≤15 x10^9^ cells/L have very strong association with death in the unadjusted model, but they might have influenced one another. Leukocytosis lost its statistical significance in causing death when the effects of other factors considered in the analyses were included: male sex, incomplete primary immunization, hoarse voice, stridor, bullneck and lymphadenopathy. Since the number of deceased patients was very small, it was hard to support more than 2 predictors in each model (all tested models can be found in S4 Data). Analyses of 2 predictors of death were shown in Models 4–8. Combination of bull neck and laboratory data (leukocytosis or thrombocytopenia) put bull neck as a strong death predictor (Model 4 or 5). Combination of incomplete immunization with leukocytosis, stridor or bull neck augmented the role of leukocytosis (AOR 63.464; Model 6), stridor (AOR 20.247; Model 7) and bull neck (AOR 29.739; Model 8) significantly, but the wide CIs merit attention.

## Discussion

A number of countries have experienced the reemergence of diphtheria, including Indonesia, which has been associated with low coverage of immunization, population migration, social-economic factors and waning immunity among the population [1, 2, 4].

During an outbreak, “panic” may set in among clinicians that can understandably lead to overdiagnosis of many patients with suspected diphtheria. Around 25% of all cases with preliminary diagnosis of suspected diphtheria were discharged from hospitals with acute tonsillitis non-diphtheria or other diagnosis. Since diphtheria cases have decreased in Indonesia, many clinicians have never encountered any patients with diphtheria with unique formation of pseudomembranes which leads to overdiagnosis of suspected diphtheria in patients with acute bacterial tonsillitis. Diagnosis of diphtheria is more of a clinical diagnosis that is supported by laboratory examination on the growth of *C*. *diphtheriae*.

Decline in cases of diphtheria after the peak in December 2017 and January 2018 may have been caused by the implementation of the Outbreak Response Immunization (ORI) program in mid-December 2017, January 2018 and July 2018.

During the outbreak in 2017 until the middle of 2018, 58.3% clinical diphtheria cases were boys (vs 41.7% were girls). The outbreak in East Kalimantan, Indonesia and other countries reported the similar proportion [8–10]. By age distribution, diphtheria most often occurs in cases <10 years old. Age distribution of diphtheria patients reflects the immunization status of a population in a given area. Most cases of diphtheria occur in primary school age children and teens. Dominant occurrence of diphtheria in children under five years old shows low coverage of basic immunization [11]. In countries with high coverage of immunization, diphtheria cases in infants and children are very low but these populations are still very susceptible in the event of an outbreak. Teen and adult populations are also susceptible to infection when there is no exposure to (naturally) circulating toxigenic bacteria and in the absence of booster immunizations [1, 12]. A prospective research in Russia in 2000 also indicated that 52% of diphtheria cases and 68% of mortality from diphtheria are in the ≤14 year age group, who have high susceptibility to mortality and complications [13].

Although a number of publication reviews reported a shift in the age group infected by diphtheria to children in older age group and adults [1, 2, 14], this research found that the 1–4 years and 5–9 years age groups still remained the dominant age group to be infected by diphtheria (Table 2; 28.6% and 37.8%, respectively) compared to children in older age groups. Prior to the diphtheria toxoid vaccine era, 40% of diphtheria cases occurred in children <5 years of age and 70% in children <15 years of age [1, 14–16]. After the vaccine era in endemic countries, cases shifted to among teens and young adults >15 years old. This change has been attributed to the low population to be covered by immunization in developing countries in which natural circulation of diphtheria helped maintain immunity among adult and older age groups [2]. In developing countries with warm climate, patients with skin diphtheria can boost immunity even without any typical respiratory diphtheria symptoms or risk [17]. In countries where diphtheria is well controlled, population immunity wanes among older age children or teens [18]. In some developed countries, adult population age groups are no longer immune and therefore depend on various implemented immunization programs [1].

This research indicated that diphtheria mortality occurred in 70% of the patients who did not receive complete primer diphtheria toxoid immunizations, and this was one of the independent predictors for fatality (AOR 12.49; 95%CI 1.615–96.64; *p*<0.05 for more than 2 predictors model, and AOR 6.603; 95%CI 1.553–28.069; *p*<0.01 for 2 predictors model). Another study in India indicated that 95% of mortality cases did not have complete immunization history although 5% of mortality also occurred in the <2 years old age group who received basic immunizations [18]. Phalkey *et al*. reported that 2 of 10 confirmed cases of diphtheria occurred in children with complete diphtheria immunizations [11], and two studies in Russia also reported that an outbreak could occur in populations with high coverage of immunizations [19, 20].

Diphtheria vaccine is very effective in preventing severe forms of diphtheria infection or mortality, but is not effective in preventing patients with mild infection or asymptomatic carriers of diphtheria [21]. Diphtheria toxoid vaccine can provide 90% protection against clinical diphtheria and 50–80% protection against myocarditis, neurological complications and death [6, 7, 13, 22]. Circulating toxigenic biotypes serves as a natural booster and can maintain the immunity of the population against infection. Antibody levels decline with age so that in some developed countries, less than 50% of the adult population are immune to diphtheria. Adult age 20–40 years old or older are the age groups with the lowest level of antibody [21, 23]. These age groups are susceptible and at risk to cause a diphtheria epidemic.

In diphtheria infection, clinical signs and symptoms, such as hoarse voice, stridor, pseudomembrane, lymphadenopathy, and bull neck are considered as predictors for fatal outcome [13, 18, 24]. More recently, hoarse voice was found in 83% of deceased children with diphtheria (OR 13.68; 95%CI 2.63–95.09; *p* 0.0001), stridor in 50%, (OR 22.67; 95%CI 4.67–117.15; p<0.0001), and bull neck in all deceased patients (*p*<0.0001) [9]. Bull neck occurred as a result of response against diphtheria infection that causes lymphadenopathy, adenitis and soft tissue edema in the surrounding region. Bull neck usually occurs in severe cases [25].

We were unable to determine the certain onset of illness, so we utilized the onset of antitoxin treatment when patients were admitted to the hospital. All deceased patients in our study received antitoxin on day 0–1 when admitted to the hospital and we were unable to prove its association with death (*p* 0.364). Each day of delay increased the likelihood of mortality outcome, in which CFR increased from 4% to 7% for those provided with antitoxin on the first day of illness compared to day 4 or day 5 after illness [13]. The scarce supply and frequent lack of availability of the antitoxin were the main reasons for not providing antitoxin treatment in time.

It is widely understood that there is no specific peripheral blood examination pattern for diphtheria infection. Leukocytosis may occur but not at very high levels and not with pathognomonic patterns. Other previously reported leukocytosis may occur with neutrophilia and higher level of *C-reactive protein* (CRP) [26] and leukocytosis level of >25 x10^9^ cells/L was associated with mortality in myocarditis diphtheria [5]. Leukocytosis may have attributed to the relatively severe condition of the patients when admitted to the hospitals.

Thrombocytopenia rarely occurs in diphtheria infection in general, except in severe infection with complications. In patients with the tendency of blood loss, decline in the thrombocyte count, increase in fibrinogen degradation product level, and increase in D-dimer level may occur [9]. Thrombocytopenia was also found in 31.3% of the children wi3th diphtheria among those admitted to the intensive care unit, and platelet count <150 x10^9^ cells/L was associated with death (OR 1.9; 95%CI 0.45–8.2; *p* 0.32) [27].

Diphtheria fatality usually has been a consequence of toxin-mediated myocarditis. In our study, myocarditis complications occurred in 45 patients of the total of 283 patients (15.9%). Myocarditis was present in all fatal cases (*p*<0.001). Diphtheria myocarditis was associated with 60–70% mortality rate and was the main cause of mortality in diphtheria infection [27]. A prospective research in Vietnam reported that 40% patients with diphtheria myocarditis had shown symptoms of cardiomyopathy on the day they were admitted to the hospital. The remaining patients who did not show symptoms were later diagnosed with cardiomyopathy during their treatment. Twelve patients of a total of 32 patients (37.5%) with cardiomyopathy died [9].

Our study limitations involved the use of the retrospective cohort method, which did not include detailed history of the onset and course of illness, contact and travel history. Not all of the myocarditis patients had complete cardiac markers examination together with electrocardiograph records. On the other hand, our study was strengthened by the large patient population from six referral hospitals across Jakarta Province and Tangerang District, Banten Province.

In conclusion, our study extends previous reports that adequate immunization is one of the major determinants of death. The appearance of classical severe clinical manifestations is still important to be recognized as the main fatal predictors. Simple laboratory investigations such as leukocyte and platelet counts did not receive due attention before, but apparently have an important role in the course of the illness. Maintenance of diphtheria antitoxin availability within country should be ensured and more referral laboratory diagnostic capabilities for *C*. *diphtheriae* culture and toxigenicity testing are needed.

## Supporting information

S1 Data Cases by month. (XLS)

S2 Data Dataset clinical and laboratory data of study subjects. (XLS)

S3 Data Age group and immunization. (XLSX)

S4 Data Modeling predictors. (XLSX)
